# Improving care for people with heart failure in Uganda: serial in-depth interviews with patients’ and their health care professionals

**DOI:** 10.1186/s13104-017-2505-0

**Published:** 2017-05-25

**Authors:** Elizabeth Namukwaya, Liz Grant, Julia Downing, Mhoira Leng, Scott A. Murray

**Affiliations:** 1 Makerere University College of Health Sciences, P.O BOX 7072, Kampala, Uganda; 2 Global Health Academy, The Usher Institute of Population Health Sciences and Informatics, The University of Edinburgh, Medical School, Teviot Place, Edinburgh, EH8 9AG UK; 3 Primary Palliative Care Research Group, The Usher Institute of Population Health Sciences and Informatics, The University of Edinburgh, Medical School, Teviot Place, Edinburgh, EH8 9AG UK

**Keywords:** Heart failure, Palliative care, Qualitative research, Patient experience, Uganda

## Abstract

**Background:**

The short prognosis of patients with advanced heart failure (HF) and the associated multidimensional distress as illustrated in literature from high income countries necessitates the integration of palliative care into the care of advanced HF patients to address these needs and improve their quality of life. These needs, which are subjective, have not been described from the patients’ and health care professionals’(HPs) view point in the Ugandan setting, a low income country with a different socio-cultural context. This study aimed at bridging this gap in knowledge by eliciting patients’ and HPs’ views of HF patients’ needs over the course of their illness to enable generalists, cardiologists and palliative care clinicians to develop guidelines to provide patient-centred realistic care in Uganda.

**Methods:**

Serial qualitative in-depth interviews were conducted with HF patients who were purposively sampled and recruited in Mulago National Referral Hospital (MNRH) until thematic saturation. In-depth interviews were conducted at three time points with intervals of 3 month between interviews over the course of their illness in the hospital and their home context. One-off interviews were conducted with HPs that manage HF in MNRH. We used a grounded theory approach in data analysis. The Uganda National Council of science and technology approved the research.

**Results:**

Forty-eight interviews were conducted with 21 patients and their carers and eight interviews with their HPs. Multidimensional needs including physical, psychological, social, spiritual and information needs were identified. These highlighted the underpinning need to have normal functioning, control, to cope and adapt to a changed life and to find meaning. Spiritual needs were less recognised by HPs than the other multidimensional needs. Information needs were commonly unmet. Patients and HPs suggested improvements in care that were congruent with the recommendations in chronic disease care and the six pillars of the WHO health systems strengthening approach.

**Conclusion:**

Management of HF in Uganda requires an approach that targets multidimensional needs, embraces multidisciplinary care and strengthens health systems which are all important tenets of palliative care.

## Background

The burden of non-communicable diseases (NCDs) is rising fast in low-income countries such as Uganda and they will be the leading cause of death by 2030 [1–3]. Cardiovascular diseases including HF contribute to 30% of NCD deaths [4–6]. Most patients with HF in Africa present with advanced disease [7]. Literature from high income countries shows that advanced HF leads to multidimensional distress and reduces people’s quality of life. HF causes high symptom burden and distress akin to that of cancer [8–11] and impaired physical functioning [12]. Its psychological impact leads to the need for emotional support [10, 12]. Ill health due to HF leads to changes in people’s social lives leading to the need for independence, [13] to have a life free of disruption [14] and for support groups [13]. In the existential dimension the effects of HF lead to the need for; peace of mind, freedom from fear of death, to be in control, dignity, respect, to find meaning and purpose in life and for faith and prayers [15–18]. Patients with HF also have needs for clear information about their illness [12, 19–21].

The prognosis of HF can be worse than that for bowel and breast cancer [22, 23]. Thus it is important to integrate palliative care in managing patients with advanced disease while continuing their HF medication. However, most palliative care services in Uganda are focused on HIV/AIDS and cancer and are not set up to help people with HF. In order to establish patient centred palliative care for people with HF, it is important to understand patients’ experiences and needs through their perspectives. There is limited peer-reviewed literature from Africa that reports this [24, 25]. This study thus aimed to describe patients’ experiences of their illness, their perspectives of their multidimensional needs over the illness course and what they and their HPs want to be improved. This will enable generalists, cardiologists and palliative care clinicians to develop guidelines to provide patient-centred realistic care in Uganda.

## Methods

### Setting

This study was conducted on the general cardiology ward in MNRH in Kampala, Uganda, and in patients’ homesteads from March 2013 to January 2014. Patients were recruited in hospital and all first interviews were conducted there. Subsequent interviews were done either at home or in hospital.

### Theoretical framework

We adopted a grounded theory approach and an interpretivist perspective and constructivist epistemology. An interpretivist perspective enables eliciting rich information related to individual perceptions of issues [26] and constructivism enables one to take into consideration multiple views that people with HF will construct of their experience influenced by their social, cultural and historical context. This is important in generating interventions that meet people’s needs in their context [27, 28].

## Methods

We conducted serial qualitative in-depth interviews with patients and their family carers, and qualitative one-off interviews with HPs. Qualitative longitudinal multi-perspective interviews were employed so as to get an in-depth understanding of the patients’ complex dynamic needs and how care could be improved from both HP and patient perspectives [29, 30].

### Sampling procedure

We purposively selected patients to represent the local demography of New York Heart Association stage 3 and 4 HF. A review of the MNRH database of HF patients performed at the start of this study showed clustering of HF around the age groups of 20–40 years and the 50–80 years. These groups also had different causes of HF, therefore sampling aimed at including patients from each of these age groups and recruitment was done until thematic saturation was achieved. HPs were purposively sampled to include those directly involved in the management of HF patients. Diversity of HPs was sought for a broad range of views.

Patients were eligible if they were 18 years or older, had 4 out of the 5 criteria of the definition of advanced HF by Metra et al. [31] and if they lived within 30 km from Kampala. Patients were excluded if they had diminished cognitive capacity to consent, if they were too ill to be interviewed, and with profound communication deficit and if they could not speak Luganda or English, which are the languages spoken by the researcher.

### Data collection procedure

Patients were interviewed during the time of hospitalisation, and were followed up monthly by mobile phone to maintain contact and relationship and to provide an alert if there was change in health status which would trigger a subsequent interview in order to capture needs at the time. Repeat interviews were conducted at 3 and 6 months thereafter if the patients’ clinical condition remained stable, and earlier if there was marked deterioration. Bereavement interviews were conducted with family carers of those patients who died before they had completed the three interviews. Interviews were conducted with HPs involved in the care of the patients. All interviews were audio recorded and field notes were written.

### Analysis

Audio recorded interviews of the HPs were transcribed verbatim. Patient interviews were transcribed and then translated into English. Transcripts were exported into QSR Nvivo software version 10 together with the field notes. Principles from Charmaz’s grounded theory [32] (line by line coding, focused coding, constant comparison and theoretical coding) were employed in analysis. The first author read through all the transcripts and re-listened to the audio interviews to confirm completeness of transcription and accuracy of translation. Three authors discussed and agreed on codes and themes that were generated from the data. QSR Nvivo software enabled organising and working on large volumes of qualitative data.

The approach to analysis of qualitative longitudinal interviews proposed by Thomson and Holland [33] as well as that proposed by Saldana were employed [34].

### Reflexivity

The researcher was reflexive on how her role as a medical doctor influenced the data generated and any role conflict issues were anticipated and a plan drawn on how to address them. Those issues that emerged during the data collection without prior anticipation were discussed with the research team and solutions were found. The researcher had debriefing sessions after the interviews that were held at the end of each week, with a member of the team with counselling skills, during the period of data generation.

### Ethical approval and consent to participate

Approval for this study was provided by the University of Edinburgh ethics committee, Mulago Hospital Research Ethics committee and the Uganda National Council of Science and Technology (Reference Numbers D/GC/178; MREC 33, SS 3083 respectively). Permission to access patients was obtained from the administration of MNRH and the ward in-charge of the cardiology ward. The participants provided written informed consent to participate in the study by signing or applying a thumb print.

## Results

A total of 48 face to face qualitative longitudinal interviews (36-patient alone, 4 paired-patient and family carer, 8 with bereaved carers), were conducted with 21 patients and 9 carers. Eight one-off interviews were conducted with 5 doctors, 2 nurses and a social worker involved in their care.

The socio-demographic characteristics of the patients are summarised in Table 1.

**Table 1:** Characteristics of those who participated in the study

Characteristic	Total
Age group	
18–20	1 (4.8%)
21–30	7 (33.3%)
31–40	3 (14.3%)
41–50	3 (14.3%)
51–60	6 (28.6%)
61–70	1 (4.8%)
Sex	
Female	15 (71.4%)
Male	6 (28.6%)
Education level	
None	5 (23.8%)
Primary	8 (38.1%)
Secondary	7 (33.3%)
Tertiary	1 (4.8%)
Marital status	
Single	8 (38.1%)
Married	6 (28.6%)
Widowed or separated	7 (33.3%)
Diagnosis	
Dilated cardiomyopathy	4 (19.0%)
Endomyocardiofibrosis	2 (9.5%)
Hypertensive heart disease	6 (28.6%)
Rheumatic heart disease	7 (33.3%)
HIV cardiomyopathy	2 (9.5%)

Most of the patients were clustered in the age groups of 21–30 years (33.3%) and 51–60 years (28.6%). Majority 15 (71.4%) were females. Eight of the participants (38.1%) only had primary level of education and five did not have any formal education (23.8%). The commonest aetiologies for HF were rheumatic heart disease and hypertensive heart disease.

Nine family carers were interviewed (seven female, two male) including four daughters, three siblings, one parent and one partner. Family carers were mainly involved in the bereavement interviews (8) and a few participated in dual interviews with their patients (3) when asked by the patient to help answer some questions.

### Patients’ perceptions of their needs and health professionals’ perceptions of patients’ needs over the course of their illness

There were similar broad categories of multidimensional needs (physical, information, psychological, social and spiritual) identified by patients and HPs over the course of the illness. These are presented below along with illustrative quotes.

### Physical needs

#### Need to control symptoms and for cure

Participants’ physical needs included the need to control symptoms, for a cure and to prevent death. The core need underlying the physical needs was to the need to return to a functioning self so as to be able to work. Symptoms were only considered significant if they impeded ability to work.

*‘I thought, how can I go home with no medicine? I cried a lot before the health workers because I knew my pain and I knew that if I go back without the treatment…… I was worried that I would die, because I had a lot of pain.’ (Patient 2, Interview 1)*

*‘I would not aim for just improvement but when I am still sick I want to be okay and cured to be able do my work.’ (Patient 20, Interview 1)*

All HPs also recognised patients’ need to have physical symptoms controlled. However, professionals emphasised symptoms related to the cardiovascular and none talked of other symptoms outside the cardiovascular system, such as pain, that patients had described.

*‘The patients come with shortness of the breath of*-*course the different degrees, um usually we don’t see people with New York Heart Association stage 1 and 2, they are either at health centers or district hospitals they come here in New York Heart Association stages 3 and 4 and those are very sick and have body swelling, pedal oedema or ascites. Some have arrhythmias usually it is our clue to what could have brought them to hospital and they need relief.’ (HP3)*

HPs also recognised the need for a good quality of life and the need for restoration of the ability to perform daily activities.

*‘Their quality of life is affected because they are limited in how much they can do, they need other people to support them for example if you have like stage 4 heart failure, the moment they get up they feel they want to sit again. So when they feel they are in trouble they come to hospital they get care in hospital which makes them feel good.’ (HP4)*

### Information needs

There was a huge need for information probably driven by patients’ need to come to terms with and cope with the illness and to be in control.

Patients required information on the cause of the illness, the meaning of their symptoms, explanation of their test results, the role of their medications and how to care for themselves.

*‘They have done several scans but have not explained to us the results they did not explain to me the echo too. I would like to know*, what *the problem is. I would like to know, what treatment I need which one I should emphasise. Explain how the treatment will help and how it works. That will be clear and I will be more convinced and will also put in personal effort.’ (Patient 8, Interview 1)*

Most patients wanted to know their diagnosis and prognosis even it was not favourable although a few preferred not to know.

*‘If you do not tell me what the disease is or that is not going to get cured because you think it will scare me, it will not stop hurting me, so I do not want to lie to me, it is better to know if I can take care of myself then I do it.’ (Carer of Patient 18, Interview 1)*

Patients’ needs for information were only acknowledged by HPs when prompted and informed that this was an important unmet need from patients’ interviews. HPs admitted that this need was not well addressed in daily health care and identified barriers they faced in giving information including heavy patient loads, HPs perceptions that patients do not understand medical information and difficulty having some conversations especially on prognosis.

‘*Patient education, I think we are not doing enough. One because may be patient volume is too much but I think two I don’t know whether it is true the assumption that patients don’t understand the information and I think they actually do.’ (HP 3)*

*‘Sometimes you may assume they know the prognosis and then they ask questions which show they think they are going to get better and then kind of like go back to their normal life, it always brings hard questions to discuss.’ (HP7)*

### Psychological needs

Psychological needs included a need for reassurance, for empathy and for attaining life goals. Psychological needs were underpinned by the need to cope with a changed life.

#### Need for reassurance

Patients needed reassurance during the diagnosis process which was a time of uncertainty and anxiety as they went through a variety of tests which sometimes did not yield any answers and during treatment when people got treatment that carried negative connotations in the community, such as oxygen (which is associated with shortening of life).

*‘I had many tests which did not show any disease, then they checked and found that I had no HIV. I cried before the health professional I thought they were not telling the truth and told him to tell me the truth because I was in a desperate situation and wanted to know the problem. I told him to tell me the truth so that I can get treatment and be able to bring up my children because it is only me who cares for them I am their only future. So he told me I cannot tell you what I have not seen.’ (Patient 5, Interview 1)*

*‘One day they wanted to take me to have oxygen and I thought, how will they put my heart on that machine and I still continue to be alive, to be sincere I was scared of it.’ (Patient 9, Interview 1)*

The above findings and need for reassurance at particular times and in situations associated with anxiety were echoed by HPs as illustrated in the quotes below:

*‘Patients come with a number of problems most of them are anxious they are getting a unique experience especially the first timers and they have no idea what is happening most of them think they are going to die.’ (HP 1)*

*‘And then um they worry a lot. Like I had a patient with um really advanced rheumatic heart disease, she had been on oxygen for a long time, and she was 21* *years, then she one time asked “doctor will I ever get off oxygen?” I have seen many of them are worried about death and then the uncertainty about the way….’ (HP7)*

#### Need for empathy

Participants needed health workers to validate their needs and give them more attention.

*‘The other thing is that they should also put themselves (the HPs) in the position of the patient especially when they are talking to them. Let them take time to know from the patient what they need.’ (Patient 8, Interview 1)*

#### Need for attaining life goals and live a normal life

Participants expressed their need to achieve life goals and dreams and were saddened when they envisaged these dreams cut short because of illness.

*‘Yes, I am very sad because all the time I have spent in school and now I just stop there without achieving what I wanted.’ (Patient 14, Interview 1)*

HPs also understood this patients’ desire to live a normal life and to fulfil their dreams such as having a family.

*‘There are a few of them who have had issues like they want to conceive but have rheumatic heart disease and mitral stenosis and we have told them the risks, it is a bother to them because if you do not have a child you want a family and we are telling them you have to first sort out the valves and the options are limited.’ (HP4)*

Other psychological needs that HPs thought were important but were not mentioned by patients included;

#### Need for counselling and emotional support

HPs identified the need for counselling services and emotional support to help patients and their families to cope with the illness effects.

*‘Yes there is need to support them socially and of course aaa emotionally by counselling something like that to help the relatives to show them love and so that the patient can accept their illness.’ (HP8)*

#### The need to address impotence and its impact on men

HPs observed that in men, there was a particular need for addressing the common problem of impotence caused by some medicines used to treat HF because it impacted on patients significantly, but patients were shy to talk about it.

*‘Impotence is very common especially among the men, we always have to ask about it. Because one of the medications. It causes impotence in men. Carvedilol I think in some men I think it causes impotence, they may not tell you but because for me I am familiar I ask them then they tell you. Sometimes you may have to get into real counselling and also find if there is an alternative medication to that yeah.’ (HP2)*

#### Need for security and to be valued

HPs observed that patients needed to feel secure and valued and argued that it was the reason why patients who were abandoned or seen as a burden by their families and those with social stressors at home chose to stay in hospital longer.

*‘We find that some do not want to go home, they become institutionalized. They feel like not going back home you discharge them and they come back after few days. Yeah, because they know they are safe here.* W*hen you go deep or when you have time for them, they can disclose that they are not wanted at home, because of that sickness which does not go. Mmh. Yeah, they become a burden at home, so they look for where they can be helped.’ (HP6)*

### Spiritual needs

Participants’ spiritual needs were underpinned by the need to find meaning in a life that had been changed by illness. They included the following needs;

#### Need to maintain hope

Participants wanted their HPs to maintain hope despite of their illness.

*‘You get strength after counselling, if a health worker comes and says you will get better, the health worker and patient carer should not let the patient lose hope. Tell us that you are at this stage of the illness when we come back for review. But some health workers when you come back to hospital tell you we are tired of you. This is very discouraging.’ (Patient 7 Interview 1)*

#### Need to find the meaning of their illness and for spiritual support

Participants experienced periods of questioning why God let the illness happen to them.

*‘So I said to God after all I have been through is this what You choose to pay me with? Is this the time You decide to give me this? You have seen wherever I have been.’ (Patient 20 interview 1)*

They often sought existential or spiritual answers to find meaning of their illness.

*‘Illness is a test and you have to go through it. In my religion it is said that every test must have a reason for its occurrence and must have an answer to it. So this is what scares me most because, whenever there is a test and you have no answer you must be scared that you have failed.’ (Patient 8, Interview 1)*

They expressed the need for spiritual support and some started searching for this by trying different beliefs.

*‘I became a born again Christian because of this illness because He says whoever is overburdened let them come to me. So when I felt overburdened I said to myself if He says ‘I am the way the Truth and life and whoever knows this will be set free’ so I decided to be saved. I was a catholic but there is no anointment there.’ (Patient 20, Interview 1)*

#### Need to re-establish a sense of purpose

Participants wanted to re-establish a sense of purpose as they began to realise that their dreams may not become a reality.

*‘I have no parents I am already disadvantaged and if I do not go to school because of the disease, how will I just sit at my aunt’s home, how will I survive in this our Uganda. It is necessary for one to go to school. I sometimes cry because of this.’ (Patient 2, Interview 1)*

#### Need to feel cared for and to be treated with respect

Participants articulated the need for being treated humanely as this fostered a sense of dignity.

*‘I want the health workers to come just as you have come here you have sat with me and asked me what my problems are, and I need them to check me and give me medicine. I feel at peace in that way and will feel that my doctor has cared for me, he or she has understood that I am a person.’ (Patient 5, Interview 1)*

#### Health professionals’ perception of patients’ spiritual needs

Only one health professional talked of HF patients having spiritual needs. This may mean that spiritual needs are less recognised by HPs or less expressed to HPs by the patients. The spiritual need identified by the health professional was the need to pray.

*‘A number of patients have told me that fine you have given me these drugs but am also going to pray, okay, and I do encourage them to pray it but I also encourage them to take their drugs. Yes.’ (HP1)*

### Social needs during the illness

Social needs stemmed from loss of function that led to other losses, and therefore the core need underpinning the social needs was the need to adapt to the new circumstances. Social needs included;

#### The need for independence and for having control

The importance of being independent and having control to participants was demonstrated by some patients preferring to have HIV (a stigmatised condition) because HIV sufferers had time to plan before they became dependent on others.

‘*Ever since I got ill I have never been able to plan anything in my life. But at least some diseases you can plan and work and save money to support you when you are weaker. You envy those with HIV because it will not come today and make you dependent tomorrow, except if you want to. You can get treatment get better and have some time to plan. But with this condition you even fail to have some basic necessities at home and also you get to the extent of begging for 500 shillings (0.15 USD).’* *(Patient 13, Interview 1)*

Participants tried to avoid being disabled by having timely review of their illness by HPs.

*‘Whenever I feel I am getting worse I can come immediately to the hospital, I don’t wait to become like porridge, I hurry and come back to hospital. I come early and get help and do not wait to become porridge; who would carry me? I would be burdening people close to me.’ (Patient 6, Interview 1)*

#### Financial needs

Financial needs were identified by both patients and HPs. Financial crises were common in the participants’ lives because of loss of income and lead to participants failing to meet their basic needs such as getting food, the required medicines and transport to hospital for medical care. These financial crises were exacerbated by the high costs of treatment which had to be purchased on a private basis because of inconsistent hospital supplies.

*‘But this illness, I no longer work, but when I was working I was well off but since this illness started it is a problem so I stopped working* …*If I only I can get food, I am starving. I would like to eat but the financial situation does not allow me.’ (Patient 21, Interview 1)*

*‘My biggest problem is poverty because all the money I get goes to buying medicines and so all the time you are poor.’ (Patient 20, Interview 1)*

#### Need for practical help and companionship

Participants who lived alone, those who were abandoned and those who increasingly became dependent because of illness required support from their carers in all areas of their activities of daily living and sanitation and needed companionship as they became isolated due to dependency and hospitalisation.

‘*I even left my job and stayed at home to take care of him to ensure that he takes his medication on time and that he gets all the care he needed.’ (Carer Patient 3, Bereavement Interview)*

*‘I am worried I do not have someone to live* with. *I live here alone no one even to make me a cup of tea.’ (Patient 18, Interview 2)*

‘*The patient asked me doctor can my child come and see me?. They miss their families when they are in hospital’. (HP7)*

#### Need to fulfill family and social roles

Participants longed to participate in their family roles especially when they had children who were young and made efforts to do so.

‘*That is my family, they are young. I cannot even wash for them or cook for them when I want to, that is how it is with this disease.’ (she says it crying) (Patient 5, Interview 2 at home)*

The other social needs of patients as identified by the HPs but not patients, included:

#### Need for better nutritional support

HPs noted that there was need for nutritional support and argued that some clinical symptoms, such as body swelling were attributed to malnutrition as well as HF.

*‘Nutrition is a big problem.. so I think that they need that support. Um colleagues of ours in South Africa, XXX and others, they have done that before and followed up heart failure patients and their nutrition that is balancing up the diet and salt, putting some protein in the diet. You see because patients are not working, they cannot raise income for themselves and even food. So they get malnourished and when you see someone you think it heart failure when it is more than that. So I think it a big component which is lacking.’ (HP3)*

#### Need to be accepted in the community

HPs also noted the need for patients to be accepted with their condition in their community and not to be associated with stigmatised conditions. The challenge of integration was particularly pertinent for those of school age who were often wrongly thought to have HIV, rather than a heart problem as HIV was thought to be the only debilitating disease in the community.

*‘Then of course like the school going children because we see a lot of rheumatics. Their challenge is these frequent visits to the clinic then actually recently some were talking about stigma. A few of them are actually stigmatized. Especially those who go to school, because you know some people don’t know that heart failure can make someone like if your heart has failed it will make you be in hospital off school for sometime. Like I have seen people been mistaken to have HIV. Whenever they come for follow up they say my classmates think I have HIV you really see they are concerned.’ (HP 4)*

### Patients’ and health professionals’ views on how care can be improved

Both patients’ and HPs suggestions regarding improvement of care were centred on two main themes, that is improving the health care system and the health care workers.

Patients’ suggestions for improving Health care workers were centred on improving the patient–health care worker interaction. Patients suggested that this could be achieved through;

- HPs improving communication by having empathy, listening and paying attention to their needs.

*‘Let them (HPs) take time to know from the patient what they need.Some health care workers are rude, or tough, but this should be changed they HPs should also put themselves in the shoes of the patient especially when they are talking to them.’ (Patient 8, Interview 1)*

(b) HPs exhibiting professional behaviour exemplified by being respectful, giving feedback on tests and treatments and involving patients and family in decision making.

*‘The most important thing is to also let the patient know what is going on. I have noted that when the HP checks you they will not tell you that this is like this he just writes, the next HP then reads and will also keep quiet.’ (Patient 3, Interview 1)*

*‘There is one health care worker, I gave her a book when she was asking me my name because I could not talk I was so breathless, she threw back the book at me and said ‘don’t you have a mouth? If she had not heard she would have helped me and asked me again. So it is not good, they need to be trained.’ (Patient 16, Interview 1)*

### HPs’ views on improving the health care workers

HPs suggestions for improving HPs were centered on improving their skills by building capacity for staff in lower health centres so that they are able to identify and manage HF with new effective drugs in the lower health centers and increasing number of staff trained in cardiology in all hospitals.

*‘Retraining of the HPs in lower hospitals and health centers to expose them to these supposedly new drugs is very important.’ (HP1)*

*‘We should increase in the staffing of our nurses to help and to train them to know the basics of heart failure so that we increase on the critical number of nurses so that they can actually help us in taking care of these patients be it in hospital or at home.’ (HP7)*

### Improving the health system

Both patients and HPs suggested that the following had to be improved in the health system;

Improving availability of medicines for HF in all hospitals.

*‘The biggest problem is that sometimes the medicines they prescribe for us are expensive and they are not available in the hospital, so the medicines should be more available especially the expensive ones, because sometimes they prescribe them and you have no money and yet probably they would be the ones to help you and it would be very good to get them from nearby hospitals.’ (Patient 14, Interview 1)*

*‘Providing these drugs is very important. It’s a challenge in those other hospitals there are no medicines there is propanolol, atenolol and aprinox once in a while. So these other drugs ACEs, good B*-*blockers they are not there.’ (HP4)*

Providing Services for psychosocial support

*‘Counselling is needed, if a HP comes and says you will get better, the HP and carer should not let the patient lose hope.’ (Patient 7, Interview 1)*

*‘There must also be now the counselling, there must be that component of what, of patient education and counselling a very strong program for that kind of you know… So, that one actually is quite a significant component of the management. Those who have chronic HF they are quite many, so you counsel the patients and the relatives so that you can get that support.’ (HP2)*

Providing information services

*‘We need to be taught. We are so badly off in that area of information we have not been told at all, we have not been taught, may be others have been taught but certainly not me.’ (Patient 16, Interview 1)*

*‘I agree we need to give information, Yes we are trying to see how we can make this happen because even like using the screen there you can demonstrate some of those things as they wait because once they go in the consultation room, the time is ….the contact is really minimal. There are too many patients over 30.’ (HP4)*

Other suggestions that patients had for improving the health system but were not mentioned by HPs included;

Making tests and investigations more financially accessible or free of charge for patients.

*‘What I would like is that when we come to hospital and they are to do like scans of the heart, they should at least reduce the costs of these investigations or not charge for them because we are told we need 50,000 shillings (15USD) for one investigation and 80,000 (24USD) for another, one may not have this amount of money at the time.’ (Carer Patient 7)*

Better coordination of care to reduce delays in the emergency room and HF clinics.

‘Y*es the delay is real you may come early and at the end of the day you are turned away, or you may have to wait the whole day to have an investigation or to be seen, you even feel worse at the end of the day.’ (Patient 20, Interview 1)*

*‘Sometimes you have a very sick person but they consider those with money and status first. Many people die in the emergency ward. They should improve these services and see the sickest first.’ (Carer Patient 18, Interview 1)*

Having other criteria for discharge of those admitted with HF other than using the length of stay in hospital.

*‘Sometimes they discharge us too early and tell us you will get better from home. You may have spent a number of days on the ward but did not get any treatment so they may discharge you based on how long you have been on the ward.’ (Patient 5 interview 1)*

HPs also had other suggestions for improving the health system that were not mentioned by patients and these included:

Improving lower health care centers in diagnosis and treatment of HF and strengthening the referral system so as to decongest the National referral hospital so that patients could be followed up near their homes.

*‘I think we need to strengthen the referral system. Level 3, 4 and regional hospitals have to function. Once they do, it’s easier to see patients there. They do not have to be physicians at those centers because once a diagnosis has been made and the patients medication is known then just like we do with HIV we should train junior doctors how to continue treatment.’ (HP3)*

Introducing screening services for early identification of heart disease so that treatment can be instituted early.

*‘But then also we have to get to community where people are not sick you know many may have subclinical heart failure but we need to address those common causes, yeah. To go and talk about them, how to prevent the rheumatic heart disease, hypertension.’ (HP2)*

Considering community and home based care services to improve follow up of patients.

*‘Then we need an outreach program related to that such that once people are sorted then you can follow up because we don’t have a follow up model. It is mainly in the HIV setting because sometimes you know we can reach them to their own homes and give them the drugs.’ (HP4)*

Investing in advanced surgical treatments for HF.

*‘The other thing we also need to invest in these those who are at the end of the… and they need surgical…. they need devices, I think we need to invest in that because at the moment we have the majority of people lying the other side because if you don’t, they will just die. They need other interventions that may be curative. But medical treatments are basically supportive to delay symptoms and slow progression.’ (HP4)*

Health education of the public for prevention of heart disease, early referral and seeking care.

*‘Then of course educating the masses of those early signs so that they may come and get sorted early. Because if we manage the biggest culprit is hypertension well we will reduce the number of people who will eventually have heart failure.’ (HP4)*

Whereas HPs suggested including palliative care services and home based care as part of comprehensive care for HF, patients’ expectations did not include home based care.

*‘There is a huge role for palliative care. You know previously we used to think palliative care is pain management, then for people who were dying. But we need it because the palliative care team will easily complement all these other units like even cardiology because we will learn how to communicate to our patients.’ (HP4)*

*‘That would be giving the HPs a lot of work, it is too hard. Can you drive to my home in Luwero it is too much. The doctor studied, you cannot move the doctor. Some of our homes are in a poor state.’ (Patient 1, Interview 1)*

Patients’ and HPs’ views on how care can be improved are summarised in Table 2.

**Table 2:** Summary of patients’ and HPs suggestions on how care for HF can be improved

Improving HPs
HPs improving communication by having empathy, listening and paying attention to their needs
HPs exhibiting professional behaviour exemplified by being respectful, giving feedback on tests and treatments and involving patients and family in decision making
Improving skills of HPs and building capacity for staff in lower health centres so that they are able to identify and manage HF with new effective drugs in the lower health centers and increasing number of staff trained in cardiology in all hospitals
Improving other factors in the health system
Improving availability of medicines, tests and investigations for HF in all hospitals.
Providing Services for psychosocial support
Better coordination of care to reduce delays in the emergency room and HF clinics
Having other criteria for discharge of those admitted with HF other than using the length of stay in hospital
Improving lower health care centers in diagnosis and treatment of HF and strengthening the referral system so as to decongest the National referral hospital so that patients could be followed up near their homes
Introducing screening services for early identification of heart disease so that treatment can be instituted early
Having community and home based care services to improve follow up of patients
Investing in advanced surgical treatments for HF that were considered curative
Health education of the public for prevention of heart disease, early referral and seeking care
Integrating palliative care in HF care

## Discussion

This study identified the multidimensional needs of Ugandan patients over the course of their illness from the patients’ perspectives and perspectives of HPs involved in their care. Patients’ physical needs included the need to control both cardiovascular and non-cardiovascular symptoms and to improve their quality of life (QOL) findings also described in literature from high income countries [10, 12, 35–37]. Patients in this study had more expectations of getting a cure for their illness than those from studies from high income countries probably because they did not have enough information on the illness prognosis. The expectation for cure changed to a more realistic expectation of improving well-being as they lived longer with illness, and if they were given more information on the illness a finding that supports Bury’s work that describes people’s responses to biographical disruption, their coping, adaptation and their expectations as changing over time and influenced by the information they have [38]. We argue that the core need underpinning physical needs was the need to as far as possible have normal physical function so as to able to do their daily work and this was equated to well-being. This finding resonates with previous studies that showed improved physical functioning as a significant determiner of QOL [18]. HPs however, only mentioned cardiovascular symptoms as the main physical needs, this may imply that HPs do not recognise and manage non-cardiovascular symptoms because they expect cardiovascular symptoms, a finding also observed by Anderson et al. [10].

One of the greatest unmet needs from patients was for information on the illness, its treatment, prognosis and how to self-care, a need also described in literature from high income countries [12, 20, 21, 39, 40]. However, patients in high income countries described receiving insufficient information, particularly compared to other illnesses such as cancer, while this study demonstrated that patients in the general cardiology service received very little information. HPs confirmed this and articulated barriers to information provision including heavy patient loads, lack of appropriate contextualized information and difficulty in breaking bad news, findings consistent with prior literature [20]. Information was particularly needed during the process of diagnosis and at those times when there was little improvement from treatments, often associated with patient anxiety. Preference for timing of giving information has been observed by other authors [41]. The underlying need for information can be understood as need to regain control over their lives and to reduce anxiety. Patients had unmet psychological and social needs including the need for reassurance, empathy, counselling, the need to be valued and for independence, the need for financial and practical help, for support to fulfil family and social roles, for nutritional support and to be accepted in community in agreement with previous findings [12, 15, 37, 42, 43]. These needs were common and available services were not designed to meet them. Both patients’ and HPs’ suggested that services should be improved to address these needs. Psychosocial needs were underpinned by the need to develop strategies to cope and adapt to the illness and to repair their sense of self which was disrupted by illness. Bury’s theory of biographical disruption describes this process of people going through different reactions until they get to the stage of coping and adaptation following disruption of their daily life and future plans [38].

Patients’ spiritual needs were found to be similar to those from previous studies and included the need for maintaining hope, for purpose, respect, and spiritual support [17, 44–46]. These needs were common among patients driven by the need to find meaning in life which had changed [47] but also because spiritual support strategies are frequently employed across Sub-Saharan Africa to cope with illness [15, 48, 49]. Uganda, like many African countries is highly religious and illness is interpreted as having a spiritual rationale as well as physical [50]. Spiritual needs were identified by only one HP which may mean that patients do not express these needs to HPs but also the biomedical approach to care and training of HPs in the study context may have influenced this. The current health professionals’ training in Uganda is based on a biomedical model where spiritual, non- physical needs are not prioritized [51, 52].

As observed by other authors, patients’ and HPs did not consistently describe the same needs in each of the different dimensions [53]. This may be because while some needs are obvious because of their severity and visibility, others remain ‘hidden’. On the other hand, HPs also described patient needs patients had not described in the interviews. This may reflect the patient/provider relationship where providers are seen to be in more dominant positions and are therefore given more information in line with what Richards and Emslie observed [54]. This highlights the importance of getting both patients’ and HPs’ views.

Patients and HPs both suggested similar major themes of where improvement was needed that is improving the HPs and the functioning of health systems. Their suggestions fit with recommendations made for improvement of the management of chronic disease in low and middle-income countries [55] and the WHO six pillars of health system strengthening as well as research from low and middle-income countries which emphasise strengthening primary care, providing information, improving the health workforce, availability of medicines and technologies and improving health financing [56, 57].

Although HPs suggested home based care as a way to improve care for those with chronic advanced HF and to improve follow up, patients were not familiar with the concept of community-based care or home care and they felt it was too much to demand of HPs. This may also reflect the low expectations of patients within the population, whose experience and interaction with the health service has been one of subservience.

### Strengths and weaknesses

This is the first published in-depth qualitative longitudinal research from Uganda that has explored the multidimensional needs of HF patients from their perspective from diagnosis to death. Rich nuanced accounts were achieved to guide patient-centred care. Most previous research in Africa on HF is quantitative and is centred on the epidemiology and treatment outcomes of HF [24] which addresses different issues. This research has also shown that serial qualitative multi-perspective interviews are feasible in a low-resource setting and a different culture. Although the sample size was relatively small, it reflected the local epidemiology of HF, so findings should be relevant to the wider population. It also reflects the geographical urban/rural divide where most patients presenting were from urban areas. The study was conducted in a tertiary hospital so there are likely to be differences from those patients in more rural areas attending dispensaries if any health-care facility at all.

## Conclusions

This study demonstrated that the current care for Ugandan patients with HF that is based on the biomedical approach to illness, that stresses management of physical needs, was insufficient to meet the multidimensional of these patients.

HPs and patients suggested ways of improving care that were congruent with the WHO six pillars of health system strengthening [55] and also consistent with models of chronic disease care [56, 57].

These findings indicate that an integrated approach to chronic disease care, which includes palliative care and which involves patient education, holistic and multidisciplinary care informed by patients’ and their HPs’ experiences should be employed. This inclusive strategy will contribute to WHO health systems strengthening in low-income countries for better patients’ experiences of care. It also contributes to the World Health Assembly (WHA) resolution [3] in 2014 which called for all countries to integrate a palliative care approach for patients with all life-threatening illnesses in all settings. This study provides much information on how this can be best done in Africa from patient, family and professional perspectives. HPs’ training in management of HF should embrace a model that is more holistic than the biomedical model. More research is needed on when the Palliative care approach should be initiated in patients with HF.

## Abbreviations

HF: heart failure
NCDs: non-communicable diseases
QOL: quality of life
HPs: health professionals
MNRH: Mulago National Referral Hospital
WHO: World Health Organization
WHA: World Health Assembly
PC: palliative care
